# A phylogenetic epidemiology approach to predicting the establishment of multi-host plant pests

**DOI:** 10.1038/s42003-025-07540-y

**Published:** 2025-01-24

**Authors:** Shannon Colleen Lynch, Edeli Reyes-Gonzalez, Emily L. Bossard, Karen S. Alarcon, Natalie L. R. Love, Allan D. Hollander, Beatriz E. Nobua-Behrmann, Gregory S. Gilbert

**Affiliations:** 1 Department of Environmental Studies, University of California Santa Cruz, Santa Cruz, CA USA; 2 Department of Plant Pathology, University of California Davis, Davis, CA USA; 3 Biological Sciences Department, California Polytechnic State University, San Luis Obispo, CA USA; 4 Negaunee Institute for Plant Conservation Science and Action, Chicago Botanic Garden, Glencoe, IL USA; 5 Institute of the Environment, University of California Davis, Davis, CA USA; 6 University of California Cooperative Extension, Irvine, CA USA

**Keywords:** Ecological epidemiology, Phylogenetics

## Abstract

Forecasting emergent pest spread is paramount to mitigating their impacts. For host-specialized pests, epidemiological models of spread through a single host population are well developed. However, most pests attack multiple host species; the challenge is predicting which communities are most vulnerable to infestation. Here, we develop a phylogenetically-informed approach to predict establishment of emergent multi-host pests across heterogeneous landscapes. We model a beetle-pathogen symbiotic complex on trees, introduced from Southeast Asia to California. The *phyloEpi* model for likelihood of establishment was predicted from the phylogenetic composition of woody species in the invaded community and the influence of temperature on beetle reproduction. Plant communities dominated by close relatives of known epidemiologically critical hosts were four times more likely to become infested than communities with more distantly related species. Where microclimate favored beetle reproduction, pest establishment was greater than expected based only on species composition. We applied this *phyloEpi* model to predict infestation risk in California using weather data and complete tree inventories from 9262 1-km^2^ grids in 170 cities. Regions in the state predicted with low likelihood of infestation were confirmed by independent monitoring. Analysts can adapt these phylogenetic ecology tools to predict spread of any multi-host pest in novel habitats.

## Abstract (web summary)

A study demonstrates how a phylogenetic epidemiology approach can be used to improve rapid responses against emergent pests, which are mostly able to attack multiple host species and spread through heterogeneous landscapes.

## Introduction

Most pathogen and insect pests attack multiple host species^1–3^, but the epidemiological models traditionally used to predict the spread of emergent pests generally assume density- or frequency-dependent dynamics expected for host specialists^4,5^. Predicting which communities are most vulnerable to emergent multi-host (or polyphagous) pests is an urgent management priority because they can cause large-scale transformations of naïve ecosystems^6^ and incur significant costs^7–10^. An epidemic develops when a virulent pathogen (and any needed vectors) encounters a population of susceptible hosts where environmental conditions are favorable. For multi-host pests, the susceptible host community can include numerous species that vary in abundance and in their competence to support pest reproduction^11–14^. Rather than the density of a single host species, it is the collective abundance of such alternative host species in a local community and their ability to support pest reproduction that determine the probability of infestation and local spread^15^. Decision-makers faced with emerging infectious diseases caused by multi-host pests require robust risk models that apply knowledge of host range, reproductive potential, and environmental requirements to plant communities of concern. Unfortunately, the very novelty of emergent pests usually means that they are not at equilibrium with their environment, and therefore, empirical data on the susceptibility and competence of local host species are usually incomplete.

**Fig. 1:**
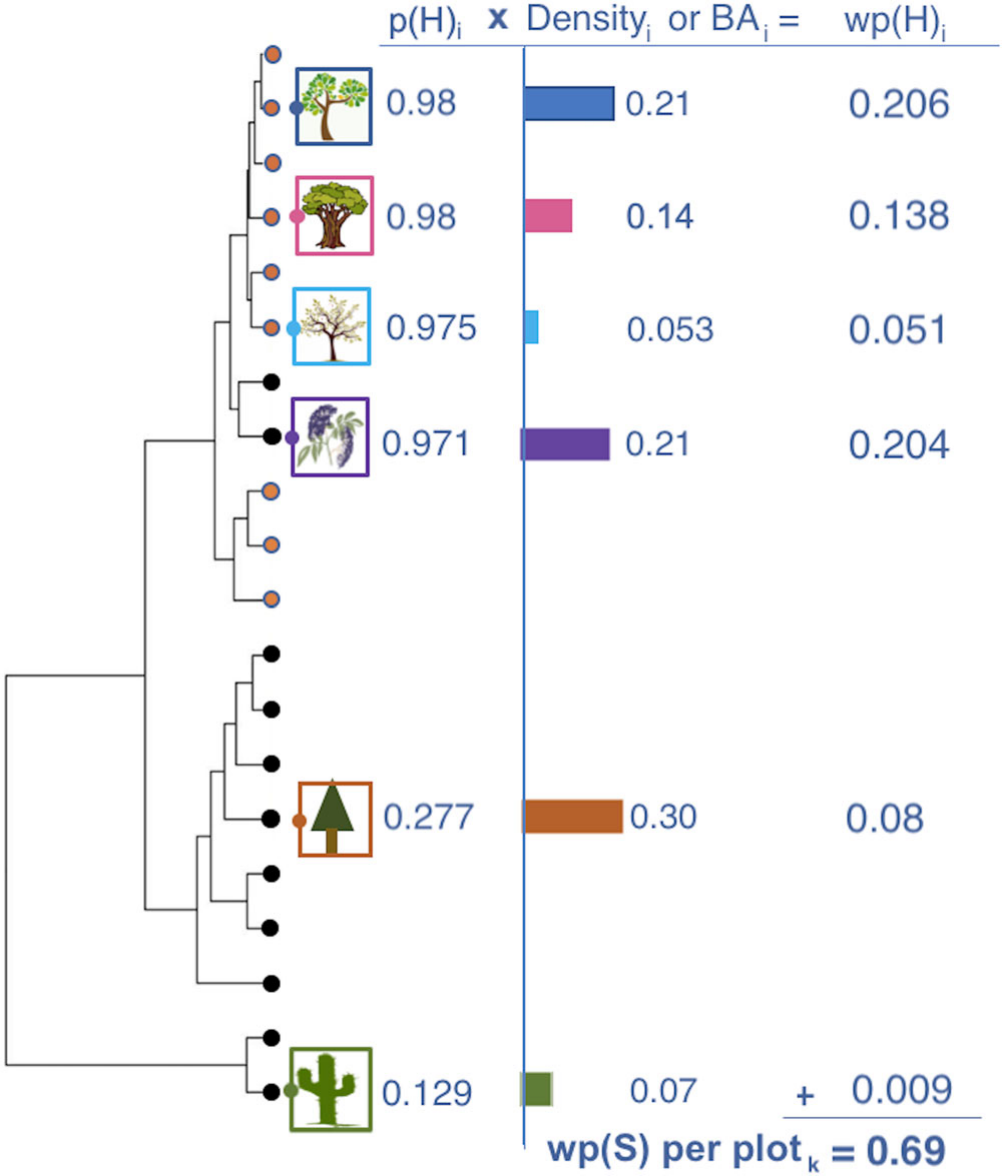
Procedure used to estimate probability that a plot would become infested by a multi-host pest. Scheme depicting the procedure used to estimate probability that a plot (*k*) would become infested by a multi-host pest, based on the interaction between phylogenetic structure and abundance (host density or basal area) of woody species in a plot. Orange tips on the phylogeny represent species that are killed-competent hosts. Here, the probability that a tree species *i* is a host (p(H)_*i*_) is a function of the phylogenetic distance of species *i* to each of 18 known killed-competent hosts *j*. The probability that tree species *i* shares the pathogen with killed-competent host *j* (p(S)_*ij*_ = antilogit (3.4–3.7 × log_10_[PD_*ij*_ + 1]) is based on the pairwise phylogenetic distance PD_*ij*_. The overall probability that tree species *i* is a host (p(H)_*i*_ = 1- ∏[1-p(S)_*ij*_]) is then the complement of the product of probabilities that tree species *i* is not susceptible to each of the 18 killed-competent host species *j*. This p(H)_*i*_ is weighted by their relative abundances (density or basal area) for each species *i* within each plot *k* (wp(H)_*i*_). The overall estimate of plot susceptibility (wp(S)_*k*_) is the sum of the weighted host probabilities within each plot *k*.

Evolutionary tradeoffs in traits that confer host susceptibility or in the ability of enemies to attack hosts produce a phylogenetic signal for host range, where closely related plant species are more likely to share pests^16^. Such a phylogenetic signal has been well documented for plant–pathogen and plant–insect interactions, including for insect-pathogen pest complexes^9,12,17^. In addition, there is a predictive phylogenetic signal of the relative impacts of pests on their hosts. For example, phylogenetic relatedness has proved capable of predicting mortality caused by multi-host animal parasites^18^; host impacts for a variety of herbivores and pathogens^19^; severity of native and non-native pests on North American tree species^20^; and the amount of herbivory-induced leaf damage on oak species (*Quercus* spp.)^21–23^. Because spread from introduced sites is an intrinsically spatial process, standard models of spread of invasive pests focus on spatial processes such as dispersal constraints as predictors of invasion^24^. In addition to dispersal abilities, realized dispersal of pests depends on the spatial availability of communities that include susceptible and competent hosts in the landscape. Given that the establishment of multi-host pests is more complex than those limited to a single host species, spatial evaluation of risk should first focus on identifying those communities that are most likely to be vulnerable based on the combination of biotic composition and abiotic conditions. Previous work has shown that phylogenetic signal in host range can be used to predict the likelihood that resident pathogens from a local plant community will spillover onto a novel host, based on previous knowledge of competent hosts^15^. Therefore, before introducing spatial processes into complex epidemiological models, we ask the reciprocal question here: can we use phylogenetic dimensions of pest host ranges to predict the likelihood that a novel multi-host pest will infest particular plant communities (Fig. 1)? We further ask how community phylogenetic structure compares in predictive importance to other factors such as environmental conditions. Once suitable habitat is identified, models of spatial spread, combined with surveillance, can be effective in describing pest spread across the landscape.

In this study, we develop and test a phylogenetically-informed approach to modeling the risk of pest establishment in wild and managed plant communities (Fig. 1). We use monitoring data from an emergent epiphytotic of Fusarium dieback–invasive shothole borers (FD–ISHB), caused by an ambrosia beetle–fungal pathogen complex that was apparently introduced to Southern California in the early 2000s from Southeast Asia^25^. The complex is a symbiotic relationship in which the ISHB beetles (*Euwallacea fornicatus* and *E. kuroshio*) depend on the *Fusarium* fungus (*F. euwallacea* and *F. kuroshium*) as a source of food, and the fungal pathogen depends on the beetles for transmission to a new host^26^. FD–ISHB has killed hundreds of thousands of apparently healthy trees in urban and wildland forests^27,28^, costing the state over $20 million^29^. New FD–ISHB introductions have emerged in South Africa^30^, where it has expanded from urban to wildland forests in eight of its nine provinces. Phylogenetic dispersion analysis on a comprehensive set of host range data^31^ shows that the strength of the phylogenetic signal is progressively more pronounced for more severely affected host species^13^; Supplementary Table 1). That is, the range of 18 host species killed by the pest-pathogen complex (i.e., killed-competent hosts) is phylogenetically narrower than the 59 additional species on which it can reproduce (i.e., competent hosts). Most recently, FD–ISHB has emerged in Western Australia^32^, where species-level empirical data on susceptibility and competence are not yet available. Phylogenetic models could be used in such situations to predict complex novel pest occurrences in different local ecosystems, serving as a critical risk assessment tool that facilitates rapid responses and forestalls damaging invasions.

We used our evolutionary understanding of the FD–ISHB host range^13^ and data from 15,000 trees in 207 0.25-ha monitoring plots to build an explanatory phylogenetic epidemiology (*phyloEpi*) model of the likelihood of pest establishment based on the phylogenetic structure of FD–ISHB host range and tree species composition in the plots (wpS; Fig. 1). Because generation time for the beetle vector is a function of temperature^33,34^, we further examined how infestation risk based on tree community composition was modified by local microclimate. To test whether these explanatory models could be used as predictive tools to prioritize actions in response to emergent diseases, we applied the model to predict infestation risk in 170 cities across California using complete urban street tree inventories^35^. We then tested the effectiveness of the model to predict FD–ISHB establishment across those cities using observational data that were not included in model development.

## Results

**Fig. 2:**
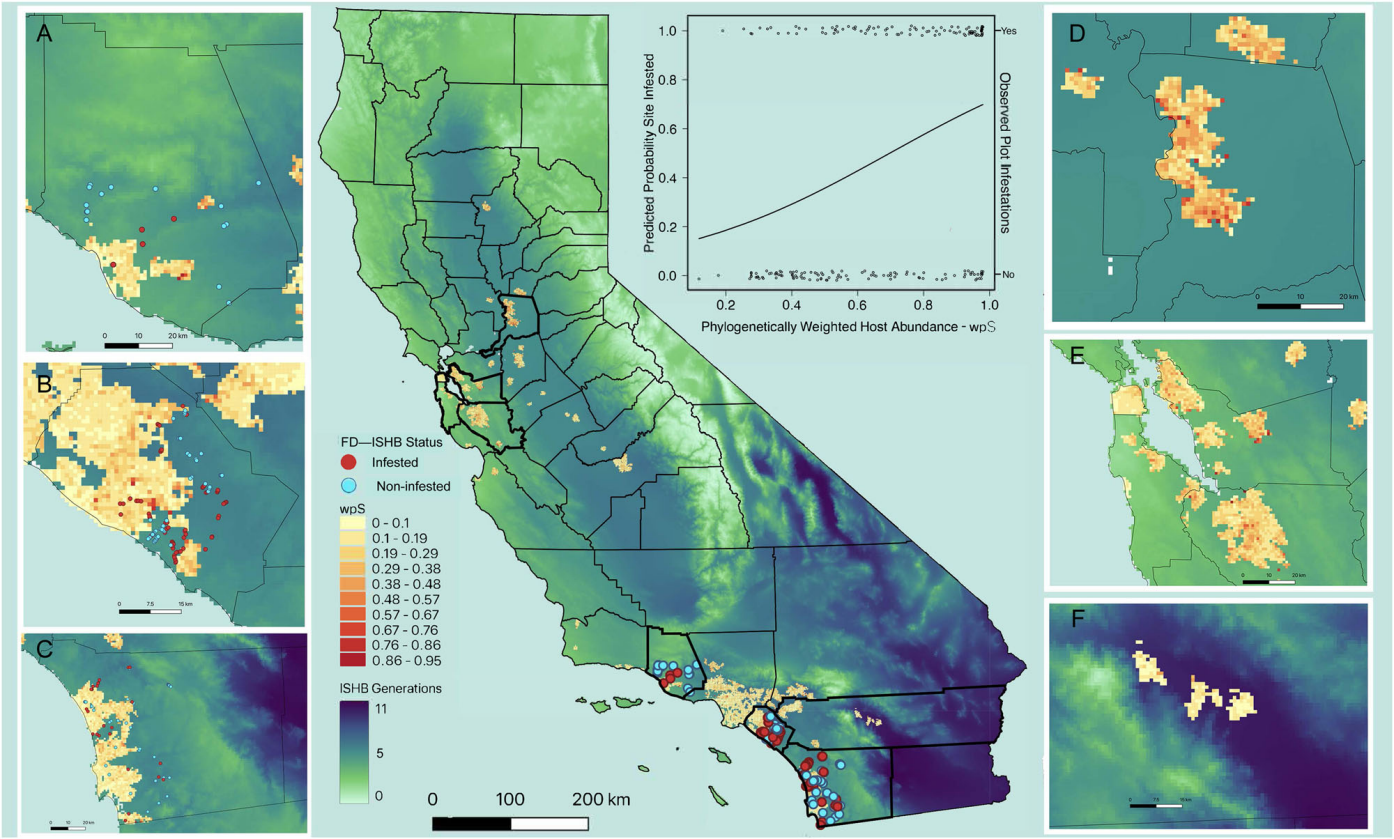
Risk map estimating plot or grid susceptibility to Fusarium dieback – invasive shothole borers (FD–ISHB). Risk map (1-km resolution) estimating plot or grid susceptibility to Fusarium dieback – invasive shothole borers (FD–ISHB) based on host species composition (yellow to red) and microclimate impact on beetle generations (light green to dark blue). The susceptibility model was developed using observational data from 207 monitoring plots (red and cyan dots) in Ventura (**A**), Orange (**B**), and San Diego (**C**) Counties (left panel) and applied to urban forests across the state (e.g., Sacramento County (**D**), Bay Area (**E**), and Palm Springs (**F**) in the right panel). Plot establishment was most strongly predicted by density-based phylogenetically weighted host abundance (wpS) of the local forest community (p(S)_*k*_ = −2.15 + 3.26 × wpS; *P* < 0.001; AIC = 231; Nagelkerke R^2^ = 0.15; Hosmer–Lemeshow Goodness-of-Fit *P* = 0.672). Grid clusters exhibit host composition-based susceptibility estimates (wpS) for urban forests with complete street tree inventories ranging from the least susceptible in yellow to the most susceptible in red. A degree-day model estimated the annual number of beetle generations that could occur within each grid; light green grids support fewer beetle generations than grids in dark blue. The current geographic infested range was determined based on regions of the state in which monitoring and surveillance had previously detected FD-ISHB.

The probability that the emergent disease complex Fusarium dieback–invasive shothole borers (FD–ISHB) establishes in a plot is a function of the phylogenetic composition of a local plant community. Plant communities with abundant close relatives of killed-competent host species are up to four times more likely to be infested than those with distantly related hosts (Fig. 2). Logistic regression revealed that neither local tree abundance nor species richness alone were predictive of beetle establishment (i.e., for total tree density *P* = 0.373; for basal area in a plot *P* = 0.838; *P* = 0.079 for species richness). Instead, the estimated probability that a site was infested increased significantly with density-based wpS (*P* < 0.001; Hosmer–Lemeshow Goodness-of-Fit Chi-squared = 1.78, *P* = 0.672; Fig. 2) and basal area-based wpS (*P* < 0.001; Hosmer–Lemeshow Goodness-of-Fit Chi-squared = 1.78, *P* = 0.672).

**Fig. 3:**
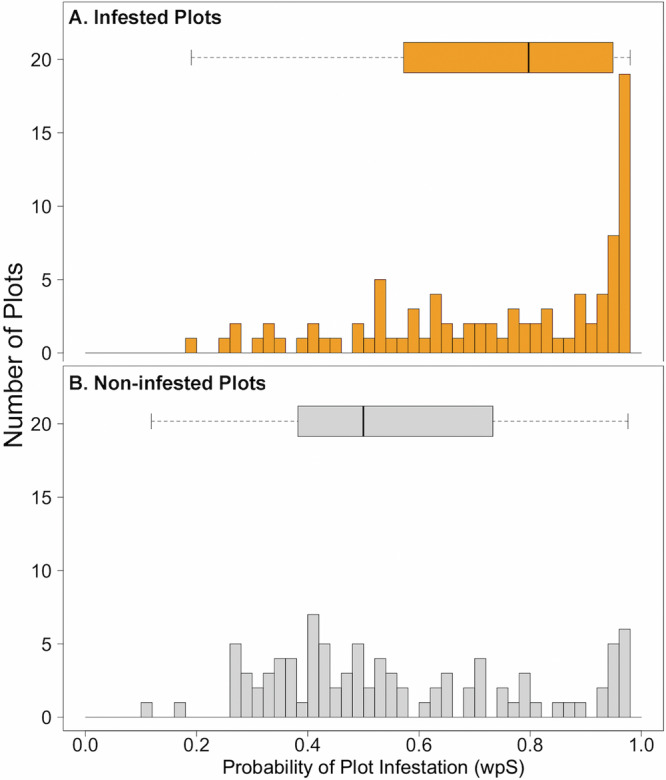
Number of observed infested and non-infested plots across abundance-weighted estimates of Fusarium dieback – invasive shothole borers (FD-ISHB) establishment in California. Number of observed (**A**) infested (*n* = 99) and (**B**) non-infested (*n* = 108) plots across abundance-weighted (host density) estimates of Fusarium dieback – invasive shothole borers (FD-ISHB) establishment in California. The predicted susceptibility of each plot is based on the local composition of woody species and their phylogenetic distance to known killed-competent host species. Whereas observed infested plots have a high predicted probability of being infested (left-skew; −0.77)), non-infested plots are expected to have a low infestation probability (right-skew; 0.82).

The abundance-weighted probability of FD–ISHB establishment for the 99 observed infested plots (median wpS = 0.80) was left skewed (skewness = −0.77; Fig. 3a and S3a), while wpS values for the 108 non-infested plots (median = 0.50) were right-skewed (skewness = 0.82; Fig. 3b and Supplementary Fig. 3b). Of the total number of plots, 75.8% of infested and only 42.6% of non-infested plots had wpS values greater than 0.60. Predictive discriminant analysis on density-based wpS estimates was able to correctly classify 74.5% of the 47 infested plots (95% CI 70.2–80.9%), and 61.5% (95% CI 61.5–69.2%) of the 39 non-infested plots. For basal area-based wpS estimates, infested plots were correctly classified 76.6% (72.3–87.2%) and non-infested plots were correctly classified 64.1% (64.1–66.7%). As expected, analysis of distance-based autocorrelation in infestation patterns suggests fine-scale patterns of spatial autocorrelation expected in invasion processes (Fig S7). Analysis of DHARMa residuals of the model showed no significant pattern that deviates from a uniform distribution, suggesting the models fit the data well (Fig S8a).

**Fig. 4:**
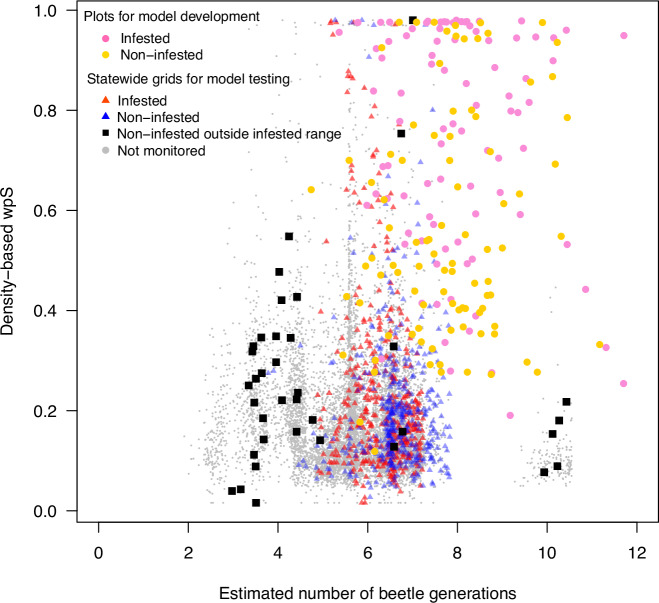
Density-based wpS estimates (phylogenetic likelihood of infestation) of plot or grid susceptibility to Fusarium dieback – invasive shothole borers (FD–ISHB) establishment in California as a function of estimated number of temperature-dependent ISHB beetle generations supported in those locations. Data from 99 infested (pink filled circles) and 108 non-infested (yellow filled circles) plots were used to parameterize the model. The model was then applied to predict infestation risk in 9262 1-km^2^ grids (gray dots) in 170 cities across California using complete urban forest inventories. A subset of 832 grids were independently monitored for FD–ISHB across its known infested (red or blue triangles) and non-infested (black squares) geographic range from 2012–2021 and used to test the model.

Further analysis indicates that the effect of temperature on infestation of plots depends on the community context: warmer places that can support more generations of beetles allow infestation of sites that are otherwise unfavorable. Logistic regression analysis detected a significant negative interaction between the effects of density-based wpS and the number of beetle generations on FD–ISHB establishment (*P* < 0.001; Nagelkerke R^2^ = 0.15; Hosmer–Lemeshow Goodness of Fit Chi-squared = 5.76, *P* = 0.98). In other words, plots expected to be non-susceptible based on species composition (density-based wpS) had a greater chance of FD–ISHB establishment where local microclimate could support more beetle generations (Supplementary Table 2). Analysis of DHARMa residuals of the model showed no significant pattern that deviates from a uniform distribution, suggesting the models fit the data well (Supplementary Fig 8b). However, the relative importance of this interaction was minor compared to the effect of phylogenetic community composition alone (Supplementary Table 2). This pattern emerged largely because microclimates are highly similar throughout the current infested range (ANOVA, *P* = 0.698), so that model development was necessarily based on locations that favor more beetle generations (Fig. 4). In addition, the interaction was not statistically significant in analyses based on basal area-based wpS estimates (*P* = 0.22). Taken together, we see that infestation was only possible where temperatures favored an adequate number of beetle generations; even where density-based wpS indicated a likely infestation, areas with fewer than five beetle generations remained non-infested. Defining the precise shape of the density-based wpS × beetle generations interaction will require validation across a broader range of host composition and microclimate conditions. Hence, our final *phyloEpi* model treats the effects of phylogenetic community composition (p(S)_k_ = −2.15 + 3.26 × wpS) and microclimate on beetle reproduction (generations = Cumulative degree days/K_ISHB_) as separate, but complementary factors, in a conceptual evaluation of site susceptibility to FD–ISHB establishment in California’s forests (Fig. 2).

**Fig. 5:**
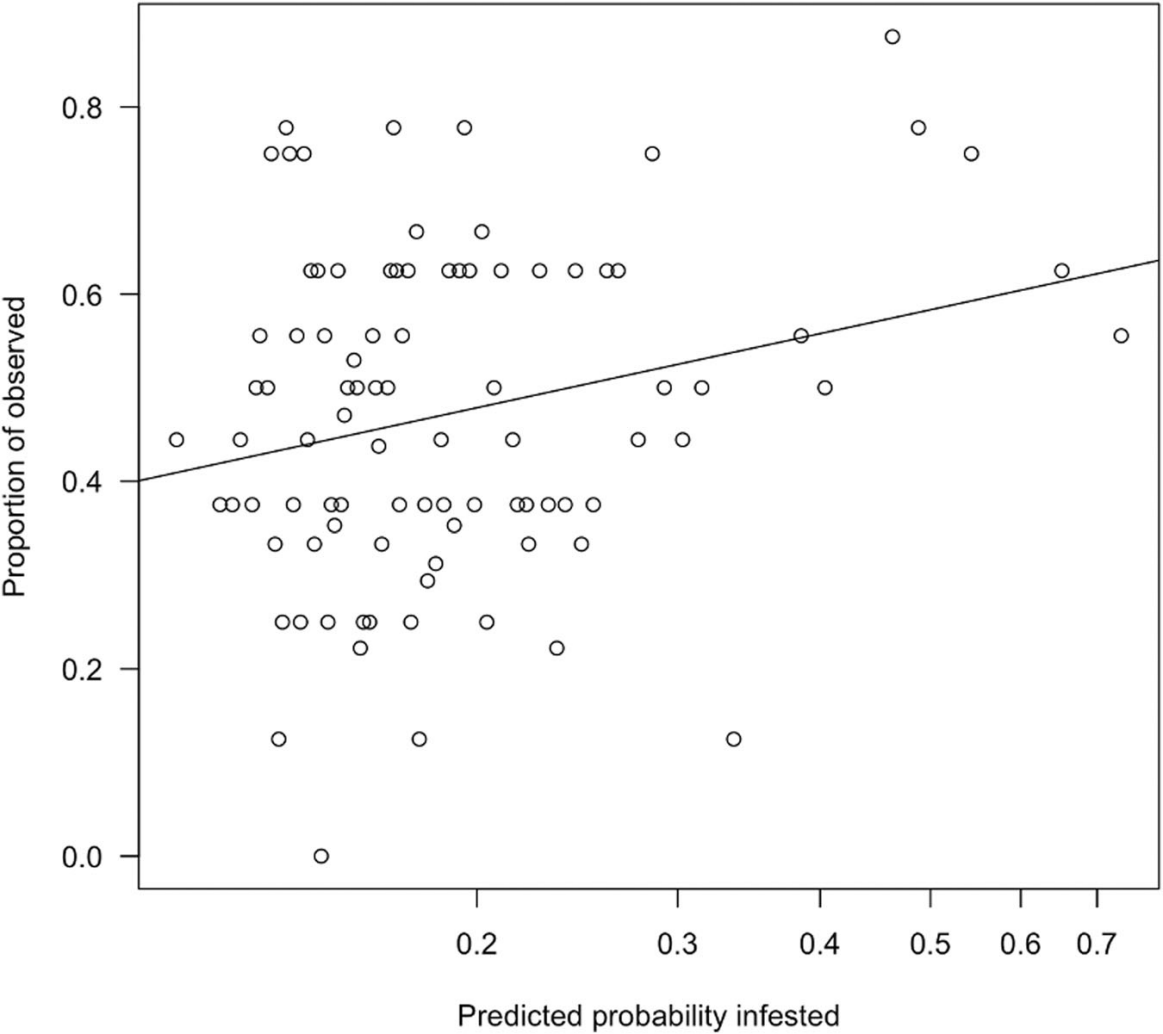
Proportion observed grids that were infested by Fusarium dieback—invasive shothole borers against predicted probability of being infested. The linear model is significant (prop(Observed) = 8.2 + 2.4 × log_10_(Predicted Prob), *P* = 0.018, R^2^ = 0.06, residual standard error (RSE) = 0.1717). Estimates were based on bins of 0.01 (*n* = 101) based on density-based wpS alone.

To test how well our model could predict susceptibility of a forest site to infestation, we used *phyloEpi* to predict the likely susceptibility of 832 urban forest grids from across California; these grids had independent FD–ISHB monitoring data and were outside our study area, so they were not used in model development (monitored grid points on Fig. 4). Most of the grids (87%) had wpS values < 0.3 and fewer than five expected beetle generations, suggesting a low probability of infestation (Fig. 4 and Supplementary Fig. 5). The *phyloEpi* model predicted observed infestation in the grids (Fig. 5; *P* = 0.019). Grids in which disease was observed generally had a greater abundance of close relatives to killed-competent hosts (Fig. 5), suggesting that phylogenetic community composition of a local plant community strongly drives pest establishment. *PhyloEpi* estimates of wpS > 0.3 were associated with 699 (24%) of the 2956 grids located outside the observed infested range, suggesting the potential for FD–ISHB establishment over a large geographic extent (e.g., Fig. 2d–f). For 32 of the 36 confirmed non-infested statewide monitored grids that were outside the zone of infestation (regions of the state outside of those areas in which monitoring and surveillance had previously detected FD-ISHB), our models forecasted a low probability of infestation, confirming the model’s predictive power across a broader range of conditions. Strikingly, the majority of monitoring locations located outside the known infested range—grids that were not included in model development—are within the ranges of wpS and generation estimates expected for non-infested sites (black squares in Fig. 4 and Supplementary Fig. 5). These patterns of model classification suggests that the relative impact of phylogenetic community composition and microclimate are important drivers for FD–ISHB establishment.

## Discussion

Decision-makers require predictive analytical tools that are robust and broadly applicable to effectively manage emergent pathogens and insect pests that attack multiple host species. Current approaches that are based on the underlying assumption that pests have narrow host ranges do not adequately address the epidemiological complexity of multi-host pests across heterogeneous plant communities. As a necessary first step to developing advanced spatial models to predict pest spread in novel habitats, we address that challenge by using the phylogenetic composition and microclimate conditions of local plant communities to predict the likelihood of multi-host pests invading those communities. The *phyloEpi* model at the core of our phylogenetic approach provides a powerful predictive tool that accounts for alternative host species driving the spread of multi-host pests across heterogeneous landscapes. As expected, plant communities with abundant species that are close relatives of known highly susceptible hosts were up to four times more likely to be infested than communities composed of distantly related hosts. The model did a better job predicting which plots would be infested (correctly classifying 72%) versus which would be non-infested (61.5% correct), most likely because currently non-infested plots could eventually become infested as the epidemic proceeds. In addition, plots deemed unfavorable for infestation based on phylogenetically-weighted species abundance were more likely to suffer FD–ISHB establishment where local microclimate could support more beetle generations; this suggests that microclimate mediates the importance of community structure on disease establishment.

We applied the *phyloEpi* model to predict infestation risk in California’s urban forests using complete street tree inventories from 9262 1-km^2^ grids in 170 cities statewide, and monitoring and weather data sets independent from data used for model development. Our model indicated that nearly one quarter of the 2956 1-km^2^ grid cells that were located outside the FD–ISHB infested range were likely to be highly susceptible to invasion. That there were no observed infestations in most of the low probability grids (32 of the 36 monitored sites) confirmed the model’s predictive power across a broader range of conditions using data sources independent of those used to parameterize the *phyloEpi* model. Together, our results demonstrate that independent of landscape and spatial factors, the establishment of novel or emergent multi-host pests through complex landscapes can be predicted through host evolutionary relationships and their abundance in a local plant community, even when extensive empirical data about susceptibility of local plant species is not yet available.

In addition to improved predictions of multi-host establishment, our approach allows us to model expected impacts. Most pest risk analyses report the relative likelihood of a species’ entry or establishment in an area, without addressing potential impacts (refs. ^24,36^; but see ref. ^37^). The model in this study predicts the probability of FD–ISHB establishment, but also incorporates two important indicators of potential damage (e.g., mortality, dieback): (1) phylogenetic signal in FD–ISHB host range, which is strongest on more severely impacted host species^13^; and (2) microclimate effects on beetle generations, which amplify impacts on hosts. Locations with high susceptibility estimates (high wpS) are therefore more likely to experience greater tree mortality. Accounting for pest impacts through a phylogenetic epidemiology approach thus fulfills the need to improve risk maps beyond potential pest distribution to also identify areas where they will likely cause the most harm^24^.

Our *phyloEpi* model could be applied to existing spatio-temporal models of pests spread (e.g., dispersal-kernel, PoPs, process-based, network) and improve their utility by integrating pest impacts^38–42^. Spread of invasive pests is inherently a spatial process, and dispersal kernels can be used to the model potential range of spread from data on the current distribution of an invasive pest^38^. The susceptible habitats identified by the phylogenetic model could be overlaid with such spatial models to identify those areas most immediately threatened with infestation. Such an extension requires data from established monitoring programs on currently infested areas.

Climate conditions provide outer envelopes around potential geographic distributions of pests, given their strong influence on pest phenology, reproduction, dispersion, and overwintering survival^43^. In this study, temperature constraints on ISHB development pointed to those regions where beetles could thrive, including locations where tree-level data are unavailable (Fig. 2). Our analysis detected that warmer places that can support more annual beetle generations could permit infestation of sites that would have been considered unfavorable based only on tree species composition. However, this predicted interaction between phylogenetic community composition and microclimate requires further validation across a broader range of host composition and microclimate conditions than where empirical data are currently available. We therefore treat these predicted patterns as tentative, but potentially useful, complementary guidance for decision makers as they evaluate where to monitor or apply appropriate preventative control measures in the immediate term. For example, some grid cells in the Central Valley and in the Bay Area have similar high probabilities of infestation risk based on host composition. Decision-makers might prioritize preventative actions in the Central Valley because those grids have more favorable temperatures for beetle development, which amplifies local susceptibility (Fig. 2d, e). Similarly, monitoring or preventative actions could also be prioritized in Palm Springs where microclimate is highly favorable for beetle establishment even though host composition suggests a lower likelihood of infestation (Fig. 2f). This approach mirrors the DAMA protocol (document, assess, monitor, act) under the Stockholm Paradigm^44^ and lays the groundwork to predicting FD–ISHB establishment under future climate change scenarios.

While climate-based risk mapping systems for pest risk analysis (e.g., CLIMEX, BIOCLIM, GARP, and NAPPFAST) provide an important first pass to setting climate-envelope bounds on future spread, they do not give land managers the resolution needed to make timely and costly site-specific decisions for host generalists^43,45–48^. Our finding that the phylogenetic composition of local communities is a better indicator of the likelihood of FD–ISHB establishment than either microclimate or host abundance alone points to the need for more high-quality tree inventory data. Sample-based forest health monitoring initiatives have produced robust and statistically reliable national forest inventory data in many countries (www.afritron.org;^49–52^, including the hundreds of thousands of periodically resampled 672-m^2^ plots in the USA Forest Inventory Analysis program^49^. However, a sample density of one plot per 24.3 km^2^ is inadequate to map tree species distributions for regional pest risk modeling, although species distribution imputation approaches (e.g., LEMMA), can be useful in filling in spatial gaps. In addition, urban forest inventories are underrepresented. The urban forest dataset used in this study includes data from publicly available municipal inventories and inventories from private arborist companies; while it represents the largest inventory of individual urban trees in the United States now publicly available^35^, the data cover only a small fraction of potential areas for FD–ISHB establishment. Given that global estimates of biological invasions represent a sizable economic burden^7–10^, anticipating where any current and future pest (e.g., spotted lantern fly) will establish and cause the most damage requires an investment in developing accessible and dependable ground-truthed host distribution datasets.

Importantly, the recent introduction of the pest means that it has not yet spread throughout the entire suitable range. Therefore, areas not currently infested include both those that are not suitable for the pest (i.e., Fig. 3b, wpS <0.55) and suitable areas that have not yet been reached (i.e., Fig. 3b, wpS > 0.55). This means that our model predictions are both conservative and tentative, but the alternative would be to wait until the pest has reached a global equilibrium across the state (which may never happen). Our purpose here is to show how areas of high susceptibility to a multi-host pest can be identified early in the process, allowing targeted surveillance and strategic management actions. For this reason, such tentative predictions hold value now.

The phylogenetic model here is applicable across systems and can be used as a robust tool to quickly determine the spread of novel pests, even as a first approximation with limited information. At a minimum, it requires a list of known hosts for a pest, to which evolutionary distances to local plant species can be calculated (i.e., p(H), Fig. 1). Because most emergent pests have been introduced from elsewhere, the local host range may not yet be known when decisions on monitoring or actions must be made. In such cases, known hosts from the geographic range of origin can be used for those calculations, with the known-host list augmented and refined as further information on locally susceptible hosts becomes available.

We developed this phylogenetic epidemiology approach to predicting multi-host pest spread out of an urgent management need for accessible decision-making tools that capture the biological realism of pests that are not host specialists^53,54^. Both range shifts driven by climate change and accidental introductions of these pests into areas outside their place of origin have resulted in novel species interactions that cause irreversible ecosystem changes with consequent ecological, social, and economic harm^55–57^. Such predictive tools are crucial for developing effective response policies because multi-host pests are more prevalent than host specialists^2,3,6^ and they are responsible for a large proportion of the most threatening emerging infectious diseases to plants, humans, and wildlife^1,53,58^. Phylogenetic signal in host range is pervasive across different groups of microbial and insect pests and can be used to predict generalist pest host ranges^15^. Here, we demonstrate that by extension, the phylogenetic composition of communities is a key predictor for the establishment of a multi-host pest. The phylogenetic ecology tools used in this study can be readily adapted to predict the establishment of any emergent multi-host pest in novel habitats. To calculate phylogenetic community composition and implement this straight forward approach, analysts can generate informative risk estimates if they have a list of known host species, plant species distribution and abundance data (i.e., density or basal area), and a host phylogeny generated using readily available tools^59–62^. Environmental data that are widely available (e.g., PRISM, CHELSA, WORLDCLIM) can then be incorporated to account for key temperature or moisture constraints on pest performance, if known. A critical next step is to integrate landscape composition and configuration factors into these phylogenetically-informed predictive models and to identify the relative importance of local and landscape features driving multi-host pest spread.

## Methods

### Site selection and plot monitoring

We established a network of 207 monitoring plots (99 infested and 108 non-infested) to measure the effect of the phylogenetic structure of tree communities and environmental conditions on the likelihood that FD–ISHB will establish in a plot, and to capture variation in pest-pathogen spread over time and space. Plots were considered infested when at least one tree within *a* ≥ 0.25 ha area was infested by the pest-pathogen complex. Plots were established between July–November 2017 in riparian habitats and oak woodlands in the coastal regions of Southern California, from Ventura County in the north (34.457666, −119.292731) to San Diego County in the south (32.555394, −117.088553) (Fig. 2 and Supplementary Data 1). Plot locations cover the range of environmental conditions in which the beetle species have been observed through extensive surveys by trained experts representing the University of California (UC) Riverside, Santa Cruz, and Davis; UC Cooperative Extension; Orange, San Diego, Los Angeles and Ventura County Agriculture; USDA Forest Service, Forest Health Protection; California Department of Forestry and Fire Protection; Disney; the Huntington Library Art Collections and Botanical Gardens; and the Los Angeles County Arboretum and Botanic Gardens (https://ucanr.edu/sites/pshb/pest-overview/ishb-fd-distribution-in-california/). Study sites were located within distinct stream courses or canyons and were chosen based on the presence of suitable tree hosts.

We established one to three monitoring plots within each site, with a minimum of 200 m between plots; vegetation composition often changed noticeably within a few hundred meters. The locations of the monitoring plots within a study site were chosen based on vegetation composition and without regard to presence or absence of the pest (Supplementary Data 1). Plot sizes were variable (0.25–2.75 ha; median = 0.27 ha) to account for variation in tree density among plant communities; plots were a minimum 50 × 50 m but were extended in low-density stands to ensure each plot included at least 50 geo-referenced trees (13, Supplementary Table 1). Plots varied in species composition and phylogenetic distances from the 77 known competent host tree species.

In each plot, we recorded attributes on every tree ≥ 5 cm diameter at standard height (d.s.h., measured at 1.37 m), and included the species and infestation status based on the presence of beetle entry holes. All trees were censused first in 2017 and then a second time in 2018. We measured hourly temperature (°C) and relative humidity (%) using iButton Hygrochron data loggers (Maxim Integrated, San Jose, CA, USA).

All together, the monitoring plots covered 7.6 ha in Ventura County, 50 ha in Orange County, and 22 ha in San Diego County (Supplementary Data 1), with 15,000 trees examined (Supplementary Table 1). Specifically, we established plots in the Oxnard Plain and Santa Clara River Valley, the Western Transverse Ranges, and the western Santa Monica Mountains in Ventura County; the Santa Ana Mountains of the Central Region and the San Joaquin hills, upper Newport Bay, and the Santa Ana River wetlands of the Coastal Region of Orange County (Aerial Information Systems 2015); and the San Luis Rey, Carlsbad, San Marcos, San Dieguito, Penaquitos, San Diego, Sweetwater, Otay, and Tijuana watersheds of the Peninsular Range in San Diego County. The network includes regions with multiple, independent pest introductions, beginning with San Diego (2014) and most recently Ventura County (2016).

Riparian habitats included both ephemerally and perennially flooded stream courses. The stream courses varied in structure, with sandy or gravelly alluvium deposited in canyons that ranged from narrow to fairly broad^63^. In addition to canyons, riparian habitat in Ventura County occurred in large valley floors of the Oxnard Plain and Santa Clara River Valley. Riparian forest physiognomy varied from woodland stands with moderately large canopy gaps in the upper tree stratum to forest stands where tree canopies overlapped between individuals so that canopy cover exceeded 100%^64^. Study sites in non-riparian coast live oak woodlands occurred along coastal foothills on north-facing slopes and shaded ravines^64^.

Stands consisted of a diversity of evergreen and winter-deciduous tree species belonging to the California broadleaf forest and woodland and southwestern North American riparian woodland, forest, and wash scrub vegetation groups (Supplementary Figs. 1 and 2)^65–67^. Combined across all sites, there were a total of 50 tree species from 25 families; 27 species were native to California (Supplementary Table 1). Common species found across most sites included western sycamore (*Platanus racemosa*), arroyo willow (*Salix lasiolepis*), red willow (*S. laevigata*), black willow (*S. gooddingii*), cottonwood (*Populus fremontii*), white alder (*Alnus rhombifolia*), and coast live oak (*Quercus agrifolia*) (Supplementary Table 1). Tree species composition varied from north to south due to the presence or absence of less dominant tree species (Supplementary Table 1). Importance values (IV) were highest for coast live oak (range 27–81%), followed by western sycamore (27–78%) and arroyo willow (25–72%) (Supplementary Table 1). Relative density of coast live oak ranged from 5 to 23%, 19–41% for arroyo willow, and 3–11% for western sycamore (Supplementary Table 1). All stands had a dense to sparse understory of shrubs including elderberry (*Sambucus nigra*) and poison oak (*Toxicodendron diversilobum*); the understory of riparian forests and woodlands also included coyote bush (*Baccharis pilularis*) and mule fat (*B. salicina*) while non-riparian oak woodlands also had toyon (*Heteromeles arbutifolia*) and California sagebrush (*Artemisia californica*).

### *PhyloEpi* model development

We tested how well the observed patterns of plot infestation could be predicted by the phylogenetic composition of the local tree community (Fig. 1). Adapting the *PhyloSusceptibility* model from Parker et al.^15^, we first calculated susceptibility (*S*) to the FD–ISHB pest-pathogen complex for tree species *i* as a function of its phylogenetic distance *PD* from a killed-competent host *j* as logit(*S*)_*ij*_ = 3.38–3.68 × log_10_(*PD*_*ij*_ + 1). The logistic regression coefficients in this equation (*β*_0_ = 3.38, *β*_1_ = −3.68) were generated using a resampling approach for the FD–ISHB pest-pathogen complex. Specifically, we randomly selected one killed-competent host species (source) and then used the *PD* from source to each plant species (target) in a logistic regression, where the response variable was 1 (susceptible) or 0 (not susceptible) and *PD* is twice the time to the most recent common ancestor in Myr. Pairwise phylogenetic distances among all tree and shrub species in California were calculated using a dated ultrametric phylogenetic tree developed by Lynch et al.^13^ and the cophenetic function in the R package Picante v. 1.2–0^66^. Logistic regression was repeated for 1000 total runs, with new random selections of source host species for each run. We calculated the median intercept and slope coefficients and the 95% confidence interval across all 1000 runs. If the 95% confidence interval of a coefficient did not include zero, it was considered significant.

Here, the probability that tree species *i* is susceptible (*p*(*S*)_*i*_) based on its phylogenetic distance from host *j* is *p*(*S*) = exp[*logit*(S)_*ij*_]/1+ exp[*logit*(S)_*ij*_]. The overall probability that tree species *i* is a host (*p*(*H*)_*i*_) is the complement of the product of probabilities that tree species *i* does not share FD–ISHB with each of the 18 killed-competent host species *j:* ${p(H)}_{i}=1-{\prod }_{j=1}^{18}\left[1-{p(S)}_{{ij}}\right]$. This *p*(*H*)_*i*_ was weighted by their relative abundances (density or basal area; Supplementary Fig. 4) for each species *i* within each plot *k* as *wp*(*H*)_*i*_ = *p*(*H*)_*i*_ * *RA*_*i*_. The overall estimate of plot susceptibility *wp*(*S*)_*k*_ is the sum of the weighted host probabilities within each plot *k*: ${{wp}(S)}_{k}={\sum }_{i=1}^{n}{{wp}(H)}_{i}$.

Estimates of plot susceptibility (wp(S)_*k*_) were compared to observed FD–ISHB establishment in plots to evaluate predictable effects of alternative hosts on pest-pathogen establishment using logistic regression and predictive quadratic discriminant analysis (QDA) on our dataset in Orange and San Diego Counties, where the beetle has been established the longest. For each analysis, those plot infestation data were randomly partitioned equally into “training” and “testing” plot data sets. We used the training data to find parameters for the discriminant model using the qda function in the MASS R package (v. 7.3–51.6), and then applied that model to the test data to predict the status of each plot (infested or not infested) based on discriminant scores. To account for classification variability on randomly partitioned data, this process was repeated 100 times for each analysis on different random sets of training and testing samples. Models at the 0.025, 0.5, and 0.975 quantile classification rates are reported as representative results.

### Annual ISHB generation estimates

Sites with microclimatic conditions more often within the optimal temperature range for ISHB development are expected to support more generations of beetles. We used a degree-day model to estimate the annual number of beetle generations that could occur within each plot as an indicator for potential propagule pressure. Observed daily minimum (T_min_) and maximum (T_max_) air temperatures in each plot were used to calculate the cumulative number of degree-days (CDD) for each day. CDD were calculated using the sine wave model^67^ with ISHB threshold temperatures for beetle emergence (T_min_ = 13 °C; T_max_ = 32 °C) determined from previous experimental work under laboratory conditions^33,34^. To estimate the annual number of ISHB generations, the CDD accumulated in 2017 and 2018 was divided by the experimentally derived thermal constant *K* for ISHB (i.e., the number of degree-days required for complete ISHB development)^33,34^. For subsequent logistical analysis of the likelihood of FD-ISHB establishment (p(FD-ISHB)), we included the density-based wpS term, the number of expected beetle generations (*Gens*), and an interaction term: p(FD-ISHB) = 8.76 × wpS + 0.5 × *Gens*–0.8 × (wpS × *Gens*)–8.06.

### *PhyloEpi* model applications

We applied the *phyloEpi* host composition-based susceptibility model (density-based wpS), which was developed and validated with our robust plot monitoring dataset, to predict urban forest susceptibility in California outside the geographic range used to train the models. We estimated susceptibility (density-based wpS) for 170 cities in California, which have complete street tree inventories in the California Urban Forest Inventory (CUFI)^35^ and are distributed across broad geographic regions where the pest-pathogen is currently found (i.e., Southern California) and where it is not (e.g., Palm Springs and Northern California’s Central Valley and San Francisco bay area) (Fig. 2). Individual tree data from the inventory were aggregated to 1-km^2^ grids across the extent of California. A total of 9262 grid cells containing 5,280,301 individual trees in 1037 species were constructed across the spatial extent of the CUFI data (Supplementary Data 2 and 3). Of the total, 2956 grids (32%) were outside the current geographic infested range. The likelihood of FD–ISHB establishment was then estimated using the total number of trees representing each species for each 1-km^2^ grid.

We tested how well *phyloEpi* predicted FD–ISHB establishment within those grids where monitoring efforts outside of our study occurred. Monitoring activities were conducted at least once from 2012–2021 in 832 (9%) of the 9262 urban forest grids used to predict density-based wpS across California (Fig. 2). The majority of data were collected during visual tree assessments within artificial boundaries where researchers had permission to survey (e.g., county parks, riparian corridors, university campuses)^68^. As such, the dataset is likely affected by some degree of spatial bias due to uneven sampling effort—a common obstacle in species distribution modeling^69,70^. Yet, with 19,161 FD–ISHB presence (9202) and absence (9959) records, it represents the most spatially and temporally comprehensive dataset currently available. We compiled FD–ISHB occurrence data from trees surveyed by trained experts representing the University of California (UC) Riverside, Davis, and Santa Cruz; UC Agriculture and Natural Resources Cooperative Extension and Integrated Pest Management; and USDA Forest Service, Forest Health Protection. Point locations for individual trees were collected using hand-held GPS devices. Grids containing confirmed infested trees were assigned “infested”, and those with only confirmed negative trees or traps outside the infested range were designated “non-infested”. A location was considered “outside the infested range” when it was not within an FD–ISHB infested county.

In addition to community-based estimates of FD–ISHB susceptibility statewide, we ran the aforementioned degree-day model to estimate the mean annual number of beetle generations across 20 years (2001–2020) within 1-km^2^ grids across California. To estimate the annual number of ISHB generations, we ran the model over a data stack giving continuous gridded estimates of daily minimum (T_min_) and maximum (T_max_) air temperature as a statewide raster for each calendar year. For this data stack, we used Daymet data in NetCDF format (https://daac.ornl.gov/DAYMET/guides/Daymet_Daily_V4.html)^71–73^. To improve computational performance given the large size of this data stack (46 GB, 365 days × 20 years × 552,000 pixels statewide), we exported the original R script to the Julia language^74^. Annual estimates were averaged across 20 years for each grid to produce a single bioclimatic map of beetle generation estimates (Fig. 2). Thus, rather than extrapolating to areas with different environmental conditions, we worked within the envelope of environmental conditions used to calibrate the model.

Ultimately, we made wpS estimates (p(S)_*k*_ = −2.15 + 3.26 × wpS) across the state only within grids that had complete tree inventory data, and removed the interaction with the effect of temperature on beetle generations (Fig. 2, wpS). We overlaid those estimates onto a separate layer of estimates of the number of beetle generations that could occur within a grid (generations = Cumulative degree days/K_ISHB_) using Daymet climate data that were available for each grid (Fig. 2, ISHB Generations). As such, we took a conservative approach in our final *phyloEpi* model statewide predictions by separating the effects of phylogenetic community composition and microclimate on beetle reproduction and treating them as separate but complementary factors in a conceptual evaluation of site susceptibility to FD–ISHB establishment in California’s forests.

### Statistics and reproducibility

The details about study design and statistics performed in this study are given in the results and methods sections. All analyses were performed using R statistical framework, with functions from the Picante v. 1.2–0, Vegan v. 1.17–8, Hmisc v. 4.3.0, phytools v. 0.6, phangorn v. 2.5.5, Geiger v. 2.0.6.2, caper v. 1.0.1 and Stats v. 2.12.2 packages (http://cran.r-project.org/).

## Supplementary information

Supplementary Information

Description of Additional Supplementary File

Supplementary Data

## Data Availability

All source data underlying the analyses and graphs presented in the main figures are available for download as Supplementary Data^1–9^ in this manuscript. Data and accompanying code used in this study can also be downloaded from the Zenodo repository (10.5281/zenodo.14600099)^75^. The 20 year-long raster stack of daily temperature minimum and maximum data to compute ISHB generation estimates within grids across California were obtained from Daymet (https://daac.ornl.gov/DAYMET/guides/Daymet_Annual_V4R1.html).

## Code availability

The R (v.4.2.2) and Julia (v.1.5.0) codes used in this study are available at the Zenodo repository (10.5281/zenodo.14600099)^75^.
