# Supplementary material for: A phylogenetic epidemiology approach to predicting the establishment of multi-host plant pests
Source: Commun Biol. 2025 Jan 24;8:117. doi: 10.1038/s42003-025-07540-y (PMC11760915; doi:10.1038/s42003-025-07540-y)
Supplement: Supplementary file 1 — Supplementary Information [file 42003_2025_7540_MOESM1_ESM.pdf]

Supporting Information for
A phylogenetic epidemiology approach to predicting the establishment of multi-host plant pests.

*Shannon Colleen Lynch, Edeli Reyes-Gonzalez, Emily Bossard, Karen S. Alarcon, Natalie L.R. Love, Allan D. Hollander, Beatriz Nobua-Behrmann, Gregory S. Gilbert

*Corresponding author: Shannon Colleen Lynch.

*Email: sclynch@ucdavis.edu

This PDF file includes:

Supplementary Figures 1 to 8
Supplementary Tables 1 to 2

Supplementary Figure, Table, and Data Captions

Supplementary Figures

Supplementary Figure 1. Number of individuals per hectare (density) of each species in California broadleaf forests and woodlands, southwestern North American riparian woodlands, southwestern North American riparian forests, southwestern North American wash scrubs, and semi-natural forests.

Supplementary Figure 2. Basal area of each species in California broadleaf forests and woodlands, southwestern North American riparian woodlands, southwestern North American riparian forests, southwestern North American wash scrubs, and semi-natural forests.

Supplementary Figure 3. Number of observed (A) infested (99) and (B) non-infested (108) plots across abundance-weighted (host basal area) estimates of Fusarium dieback – invasive shothole borer plot establishment in California. The estimated predicted susceptibility of each plot is based on the local phylogenetic composition of woody species. Whereas observed infested plots are expected to have a high probability of being infested (left-skew), non-infested plots are expected to have a low infestation probability (right-skew). The frequency of observed infested and non-infested plots across wp(S) estimates indicates that the community context can predict a multi-host infestation.

Supplementary Figure 4. Histograms of raw density, basal area, and density- and basal area-based wpS calculated values across study plots.

Supplementary Figure 5. Basal area-based wpS estimates of plot or grid susceptibility to FD–ISHB establishment in California as a function of estimated number of temperature-dependent ISHB beetle generations supported in those locations. Data from 99 infested (pink filled circles) and 108 non-infested (cyan open circles) plots were used to parameterize the model. The model was then applied to predict infestation risk in 9,262 1-km2 grids (gray dots) in 170 cities across California using complete urban forest inventories. A subset of 832 grids were independently monitored for FD–ISHB across its known infested (red or blue triangles) and non-infested (black squares) geographic range and used to test the model.

Supplementary Figure 6. Diagnostic plots of the linear regression model presented in Figure 5.

Supplementary Figure 7. DHARMa Moran's I test for distance-based autocorrelation of the density-based wpS logistic regression analysis for beetle establishment in a plot. As expected, analysis of distance-based autocorrelation in infestation patterns suggests fine-scale patterns of spatial autocorrelation expected in invasion processes (observed = 0.134, expected = -0.005, sd = 0.023, p-value = 8.506e-10). Basal area-based spatial autocorrelation checks of the logistic regression analysis yielded similar results (data not shown).

Supplementary Figure 8. Residual checks of the logistic regression models based on density-based wpS alone (A) and its interaction with annual beetle generations (B). Basal area-based models yielded similar results (data not shown).

Supplementary Tables

Supplementary Table 1. Host type, relative frequency (F), density (D), basal area (BA), and importance value (IV; [relative frequency + relative density + relative basal area]/3) of tree species ≥ 1.37 m tall and ≥ 5 cm d.s.h. in Ventura, Orange, and San Diego counties. Non-competent hosts (NC) are either attacked by the beetle species alone (A) or attacked by beetles and colonized by *Fusarium* spp. (F). Competent hosts (C) support beetle-pathogen reproduction and are killed (K) or not killed (NK) when attacked.

Supplementary Table 2. Output from the logistic regression testing the main effects of phylogenetically weighted host density (wpS), number of beetle generations (gens) and their interaction on FD–ISHB plot establishment. The regression takes the form logit(S) = β0 + (β1 x wpS) + (β2 x gens) + (β3 x [wpS x gens]) with the complementary log-log link function

(AIC=229.68).

Supplementary Data

Supplementary Data 1. Vegetation group, plot size, tree basal area (BA) and density, attacked tree density in 2017 and 2018, species richness, density- and basal area-based phylogenetically weighted susceptibility estimates, and ISHB generation estimates for each plot in Ventura, Orange, and San Diego Counties. Plots are presented in order from northwest to southeast within each county and vegetation group (California Broadleaf Forest & Woodland [BFW]; Semi-natural [SN]; and Southwestern North American Riparian Woodland, Forest, or Wash Scrub [RW, RF, or WS]).

Supplementary Data 2. Total number of grids with FD–ISHB grid susceptibility estimates within each city in California.

Supplementary Data 3. Urban forest tree species list used in this study to estimate FD–ISHB site susceptibility.

Supplementary Data 4. ISHB generation estimates using degree-day models and wPS estimates using individual tree data from the California Urban Forest Inventory, aggregated to 1-km^2 grids in 170 cities across the extent of California.

Supplementary Data 5. Subset of 832 grids that were independently monitored for FD–ISHB across its known infested and non-infested geographic range and used to test the model.

Supplementary Data 6. Phylogenetic distance matrix of all potential species ISHB could encounter calculated using a dated ultrametric phylogenetic tree developed by Lynch et al. (2021).

Supplementary Data 7. Fusarium dieback-invasive shot hole borer host traits as described in Lynch et al. (2021).

Supplementary Data 8. Species density by plot matrix used to calculate wpS.

Supplementary Data 9. Species basal area by plot matrix used to calculate wpS.

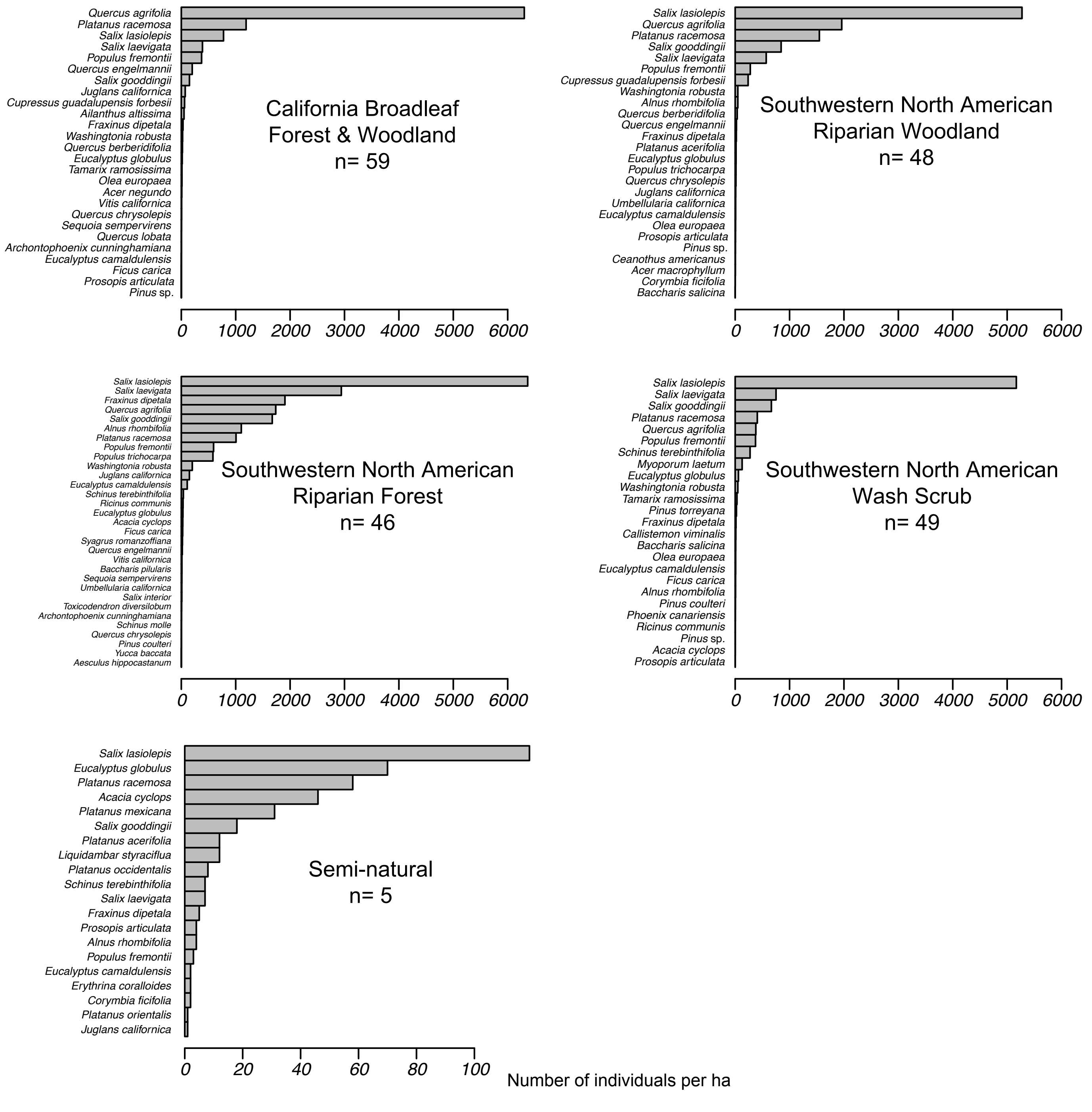

Supplementary Figure 1. Number of individuals per hectare (density) of each species in California broadleaf forests and woodlands, southwestern North American riparian woodlands, southwestern North American riparian forests, southwestern North American wash scrubs, and semi-natural forests.

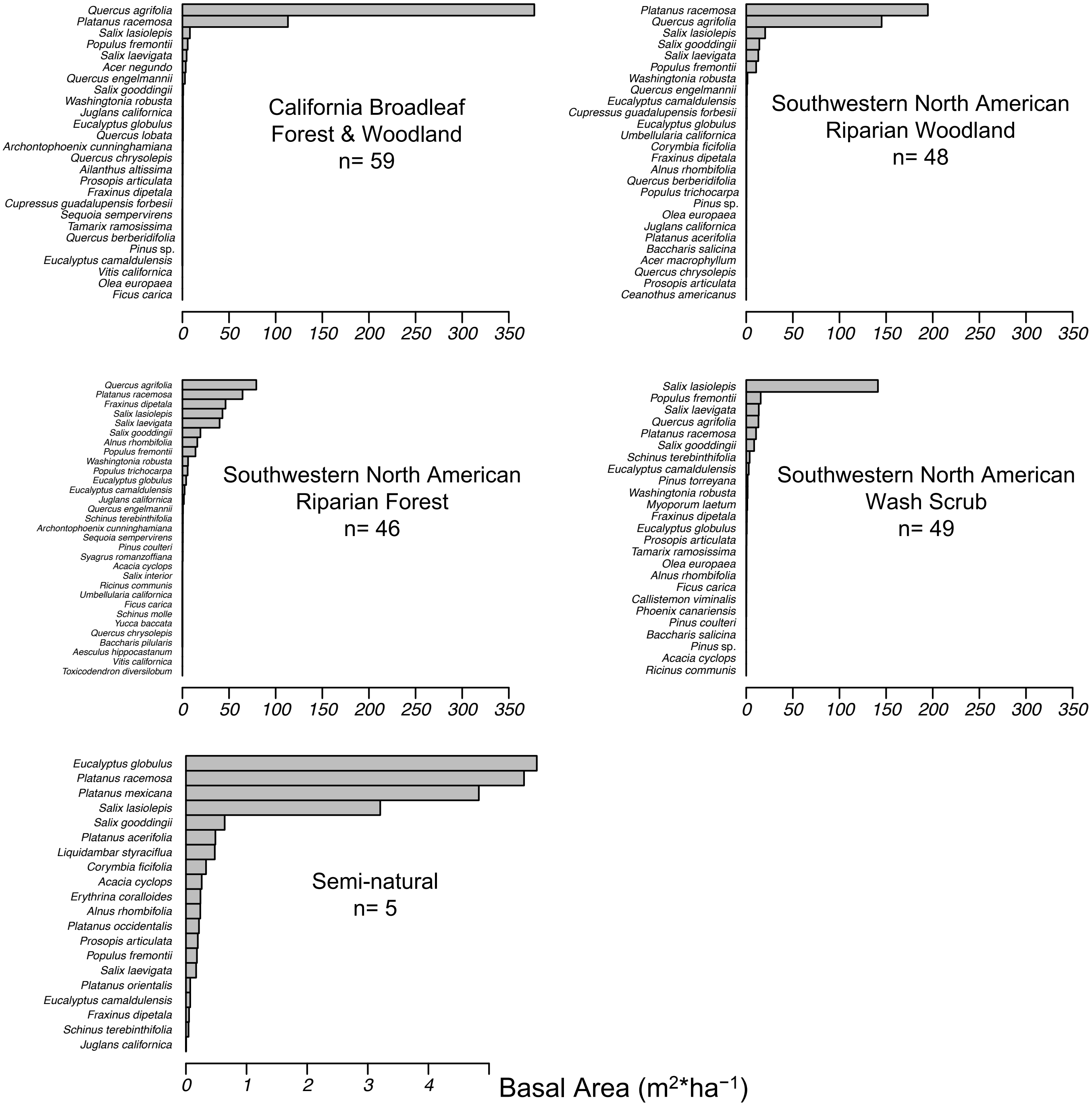

Supplementary Figure 2. Basal area of each species in California broadleaf forests and woodlands, southwestern North American riparian woodlands, southwestern North American riparian forests, southwestern North American wash scrubs, and semi-natural forests.

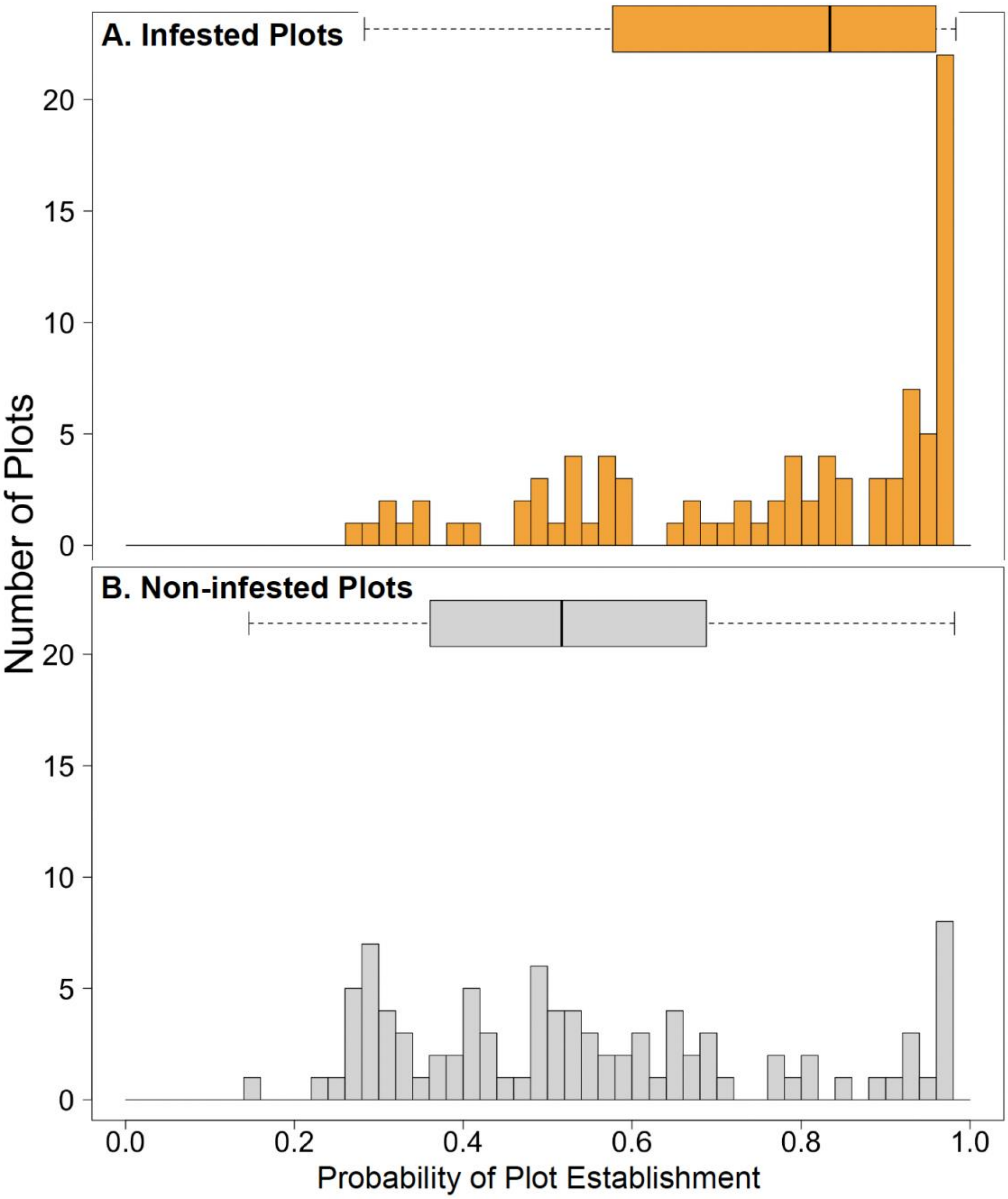

Supplementary Figure 3. Number of observed (A) infested (99) and (B) non-infested (108) plots across abundance-weighted (host basal area) estimates of Fusarium dieback – invasive shothole borer plot establishment in California. The estimated predicted susceptibility of each plot is based on the local phylogenetic composition of woody species. Whereas observed infested plots are expected to have a high probability of being infested (left-skew), non-infested plots are expected to have a low infestation probability (right-skew). The frequency of observed infested and non-infested plots across wp(S) estimates indicates that the community context can predict a multi-host infestation.

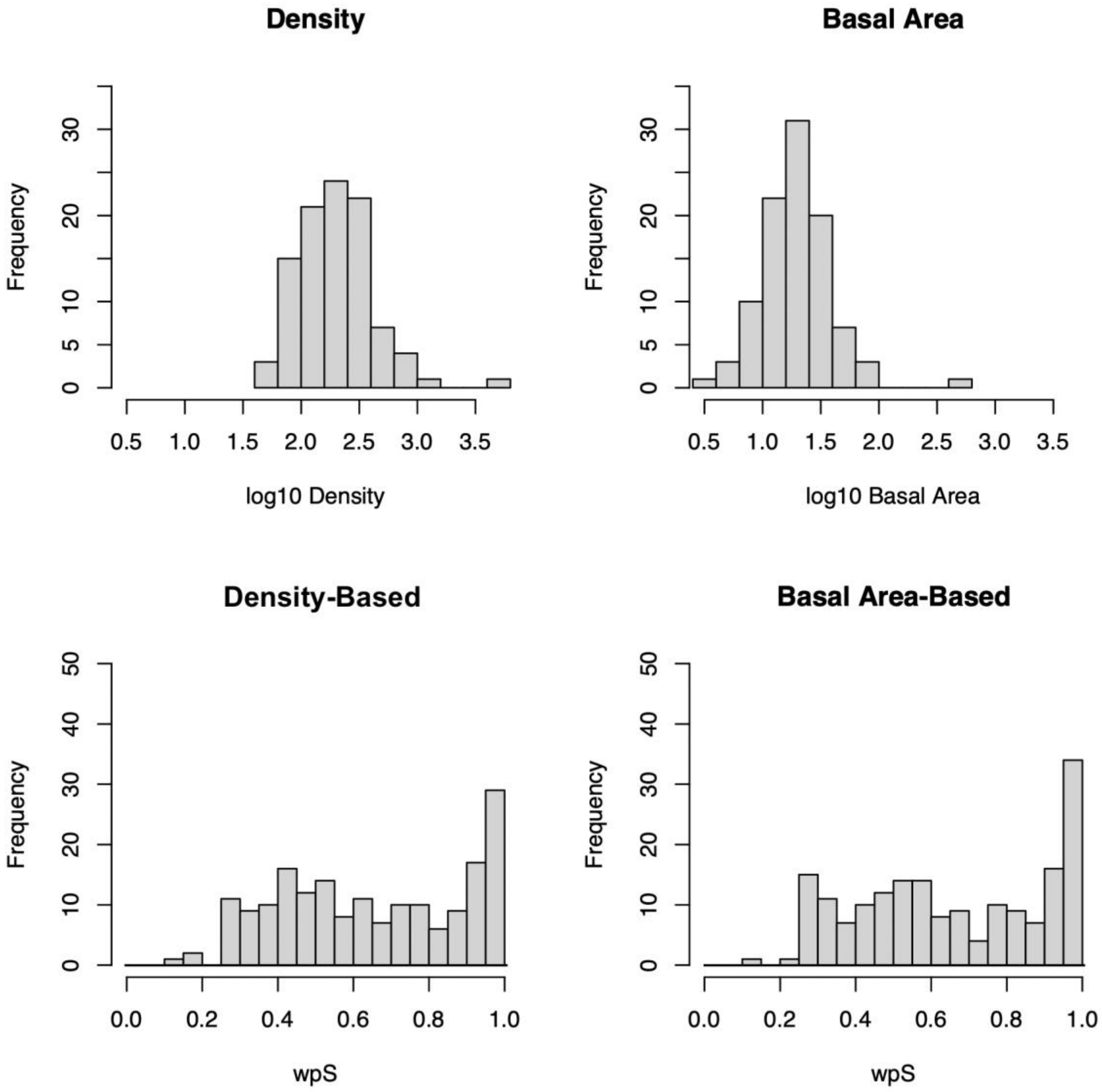

Supplementary Figure 4. Histograms of raw density, basal area, and density- and basal area-based wpS calculated values across study plots.

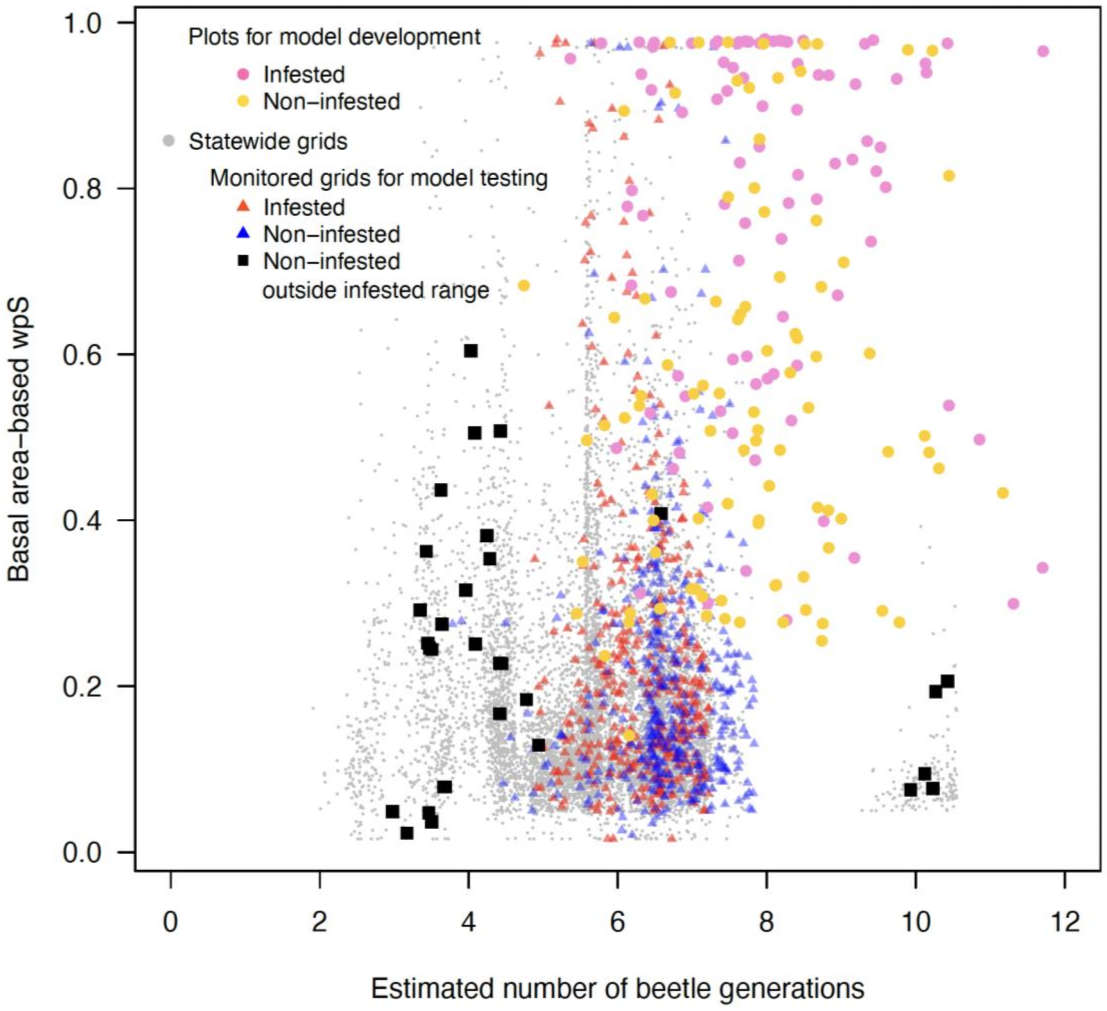

Supplementary Figure 5. Basal area-based wpS estimates of plot or grid susceptibility to FD–ISHB establishment in California as a function of estimated number of temperature-dependent ISHB beetle generations supported in those locations. Data from 99 infested (pink filled circles) and 108 non-infested (cyan open circles) plots were used to parameterize the model. The model was then applied to predict infestation risk in 9,262 1-km^2 grids (gray dots) in 170 cities across California using complete urban forest inventories. A subset of 832 grids were independently monitored for FD–ISHB across its known infested (red or blue triangles) and non-infested (black squares) geographic range and used to test the model.

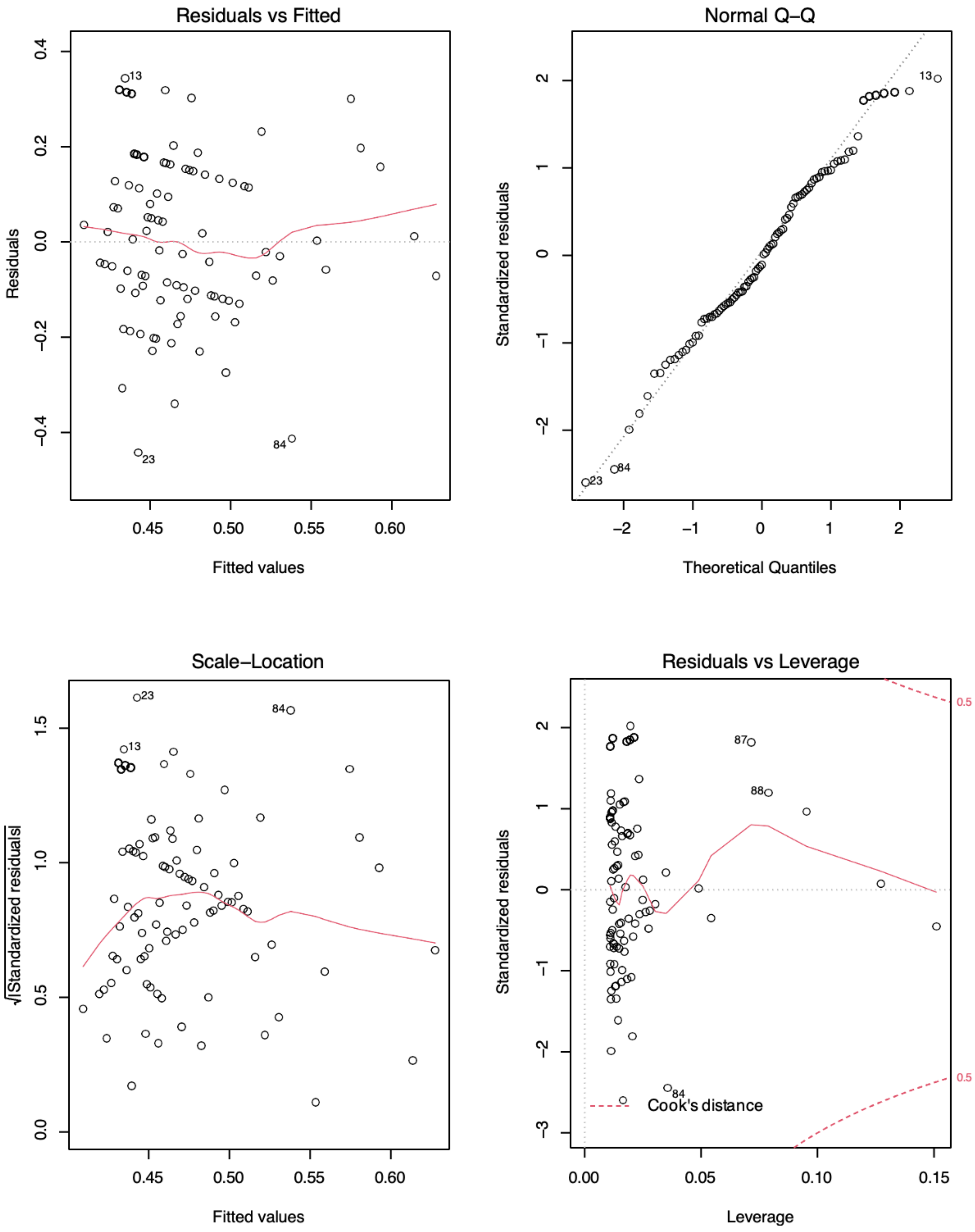

Supplementary Fig**ure** 6. Diagnostic plots of the linear regression model presented in Figure 5.

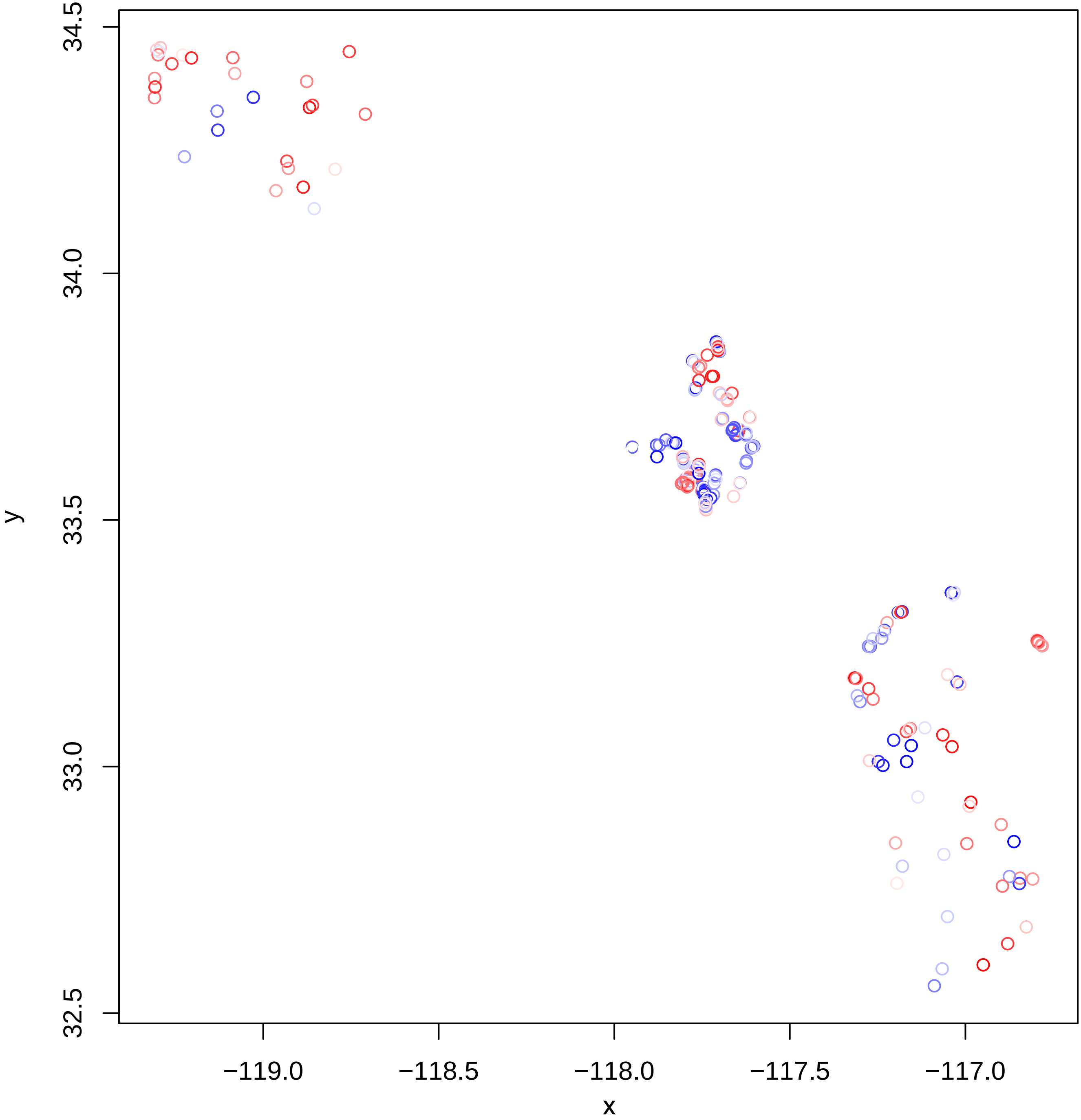

Supplementary Figure 7. DHARMa Moran's I test for distance-based autocorrelation of the density-based wpS logistic regression analysis for beetle establishment in a plot. As expected, analysis of distance-based autocorrelation in infestation patterns suggests fine-scale patterns of spatial autocorrelation expected in invasion processes (observed = 0.134, expected = -0.005, sd = 0.023, p-value = 8.506e-10). Basal area-based spatial autocorrelation checks of the logistic regression analysis yielded similar results (data not shown).

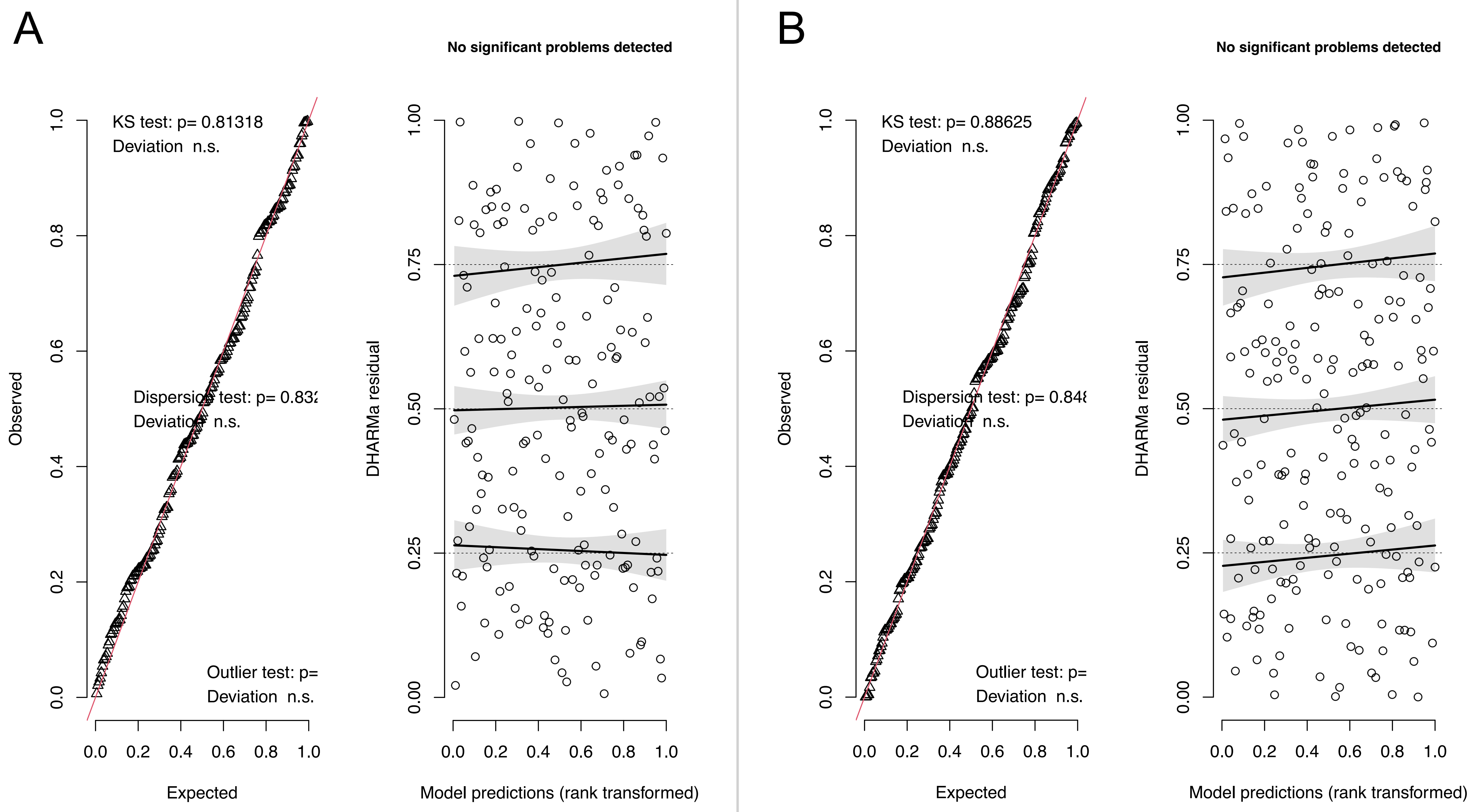

Supplementary Figure 8. Residual checks of the logistic regression models based on density-based wpS alone (A) and its interaction with annual beetle generations (B). Basal area-based models yielded similar results (data not shown).

Supplementary Table 1. Host type, relative frequency (F), density (D), basal area (BA), and importance value (IV; [relative frequency + relative density + relative basal area]/3) of tree species ≥ 1.37 m tall and ≥ 5 cm d.s.h. in Ventura, Orange, and San Diego counties. Non-competent hosts (NC) are either attacked by the beetle species alone (A) or attacked by beetles and colonized by *Fusarium* spp. (F). Competent hosts (C) support beetle-pathogen reproduction and are killed (K) or not killed (NK) when attacked.

		Host Type			Ventura				Orange				San Diego			
Family	Species	Non-Host	NC	C	F	D	BA	IV	F	D	BA	IV	F	D	Basal Area	IV
Fagaceae	*Quercus agrifolia*			NK	39.3	5.6	34.7	26.5	172.7	22.8	47.8	81.1	105.6	20.7	32.0	52.7
Platanaceae	*Platanus racemosa*			K	50.0	2.6	27.3	26.6	193.2	10.5	30.4	78.0	113.9	6.6	18.6	46.4
Salicaceae	*Salix lasiolepis*			K	46.4	19.4	10.5	25.4	165.9	40.5	8.2	71.5	138.9	24.0	23.2	62.0
Salicaceae	*Salix gooddingii*			K	7.1	0	0	2.4	104.6	6.1	2.5	37.7	80.6	10.0	3.9	31.5
Salicaceae	*Salix laevigata*			K	50.0	10.8	10.2	23.7	102.3	5.1	2.3	36.5	119.4	8.1	6.6	44.7
Caprifoliaceae	*Sambucus nigra*	NH			28.6	0.4	0.6	9.9	95.5	1.6	1.2	32.8	19.4	0.4	0.2	6.7
Salicaceae	*Populus fremontii*			K	10.7	0.4	1.5	4.2	31.8	2.6	1.9	12.1	47.2	5.0	4.9	19.0
Betulaceae	*Alnus rhombifolia*			NK	17.9	4.8	4.0	8.9	29.6	1.2	1.4	10.7	5.6	0.1	0	1.9
Anacardiaceae	*Washingtonia robusta*		A		-	-	-	-	22.7	1.2	1.0	8.3	11.1	0.2	0.3	3.9
Oleaceae	*Fraxinus dipetala*		A		7.1	0.1	0.1	2.5	18.2	2.7	0.3	7.1	38.9	6.9	7.7	17.8
Myrtaceae	*Eucalyptus globulus*	NH			3.6	0	-	1.2	15.9	0.5	0.6	5.7	22.2	0.4	1.1	7.9
Anacardiaceae	*Schinus terebinthifolia*		F		7.1	0.1	0.1	2.4	13.6	1.0	0.4	5.0	8.3	0.3	0.2	2.9
Myrtaceae	*Eucalyptus camaldulensis*	NH			3.6	0.5	0.8	1.6	13.6	0.2	0.6	4.8	2.8	0	-	0.9
Cupressaceae	*Cupressus guadalupensis*	NH			-	-	-	-	9.1	1.2	0.1	3.5	-	-	-	-
Platanaceae	*Platanus acerifolia*			K	-	-	-	-	6.8	0.1	0.1	2.3	2.8	0	0	1.0
Moraceae	*Ficus carica*			NK	-	-	-	-	6.8	0.1	0	2.3	2.8	0	-	0.9
Oleaceae	*Olea europaea*		F		3.6	0	-	1.2	6.8	0.1	0	2.3	5.6	0	-	1.9
Fagaceae	*Quercus chrysolepis*			NK	3.6	0	0	1.2	6.8	0.1	0.1	2.3	2.8	0	-	0.9
Pinaceae	*Pinus* sp.	NH			-	-	-	-	6.8	0	0	2.3	2.8	0	0	0.9
Asteraceae	*Baccharis salicina*			NK	-	-	-	-	6.8	0.1	-	2.3	-	-	-	-
Juglandaceae	*Juglans californica*	NH			35.7	1.2	1.6	12.9	6.8	0	0	2.3	5.6	0	0	1.9
Scrophulariaceae	*Myoporum laetum*		F		-	-	-	-	4.6	0.5	0.2	1.7	-	-	-	-
Arecaceae	*Syagrus romanzoffiana*	NH			-	-	-	-	4.6	0.1	0.1	1.6	-	-	-	-
Fagaceae	*Quercus engelmannii*			NK	7.1	0.3	0.3	2.6	4.6	0.1	0	1.6	33.3	0.9	0.6	11.6
Euphorbiaceae	*Ricinus communis*			K	3.6	0.1	-	1.2	4.6	0.1	0	1.6	-	-	-	-
Myrtaceae	*Corymbia ficifolia*			NK	-	-	-	-	4.6	0	0.1	1.6	-	-	-	-
Altingiaceae	*Liquidambar styraciflua*			K	-	-	-	-	4.6	0.1	0.1	1.6	-	-	-	-
Platanaceae	*Platanus mexicana*			NK	-	-	-	-	2.3	0.1	0.6	1.0	-	-	-	-
Fabaceae	*Acacia cyclops*			NK	-	-	-	-	2.3	0.1	0	0.8	5.6	0.3	0	2.0
Salicaceae	*Populus trichocarpa*			K	17.9	3.3	4.2	8.4	2.3	0.1	0	0.8	-	-	-	-
Lauraceae	*Umbellularia californica*		F		7.1	0.1	0.1	2.4	2.3	0	0.1	0.8	-	-	-	-
Fagaceae	*Quercus berberidifolia*	NH			3.6	0	0	1.2	2.3	0.1	0	0.8	5.6	0.2	0	1.9
Myrtaceae	*Callistemon viminalis*		A		-	-	-	-	2.3	0.1	-	0.8	-	-	-	-

		Host Type			Ventura				Orange				San Diego			
Family	Species	Non-Host	NC	C	F	D	BA	IV	F	D	BA	IV	F	D	Basal Area	IV
Fabaceae	*Prosopis articulata*			NK	3.6	0	0.2	1.3	2.3	0	-	0.8	5.6	0	0.1	1.9
Fabaceae	*Erythrina coralloides*			NK	-	-	-	-	2.3	0	0	0.8	-	-	-	-
Vitaceae	*Vitis girdiana*	NH			-	-	-	-	2.3	0	-	0.8	5.6	0.1	-	1.9
Platanaceae	*Platanus occidentalis*			K	-	-	-	-	2.3	-	0	0.8	2.8	0	0	1
Sapindaceae	*Acer macrophyllum*			K	-	-	-	-	2.3	0	-	0.8	-	-	-	-
Sapindaceae	*Aesculus hippocastanum*	NH			-	-	-	-	2.3	0	-	0.8	-	-	-	-
Rhamnaceae	*Ceanothus americanus*	NH			-	-	-	-	2.3	0	-	0.8	-	-	-	-
Platanaceae	*Platanus orientalis*			K	-	-	-	-	2.3	-	0	0.8	-	-	-	-
Asparagaceae	*Yucca baccata*	NH			-	-	-	-	2.3	0	-	0.8	-	-	-	-
Tamaricaceae	*Tamarix ramosissima*			NK	-	-	-	-	-	-	-	-	16.7	0.3	0.1	5.7
Pinaceae	*Pinus coulteri*	NH			-	-	-	-	-	-	-	-	5.6	0.1	0.1	1.9
Salicaceae	*Salix interior*	NH			-	-	-	-	-	-	-	-	5.6	0.1	0	1.9
Anacardiaceae	*Toxicodendron diversilobum*	NH			-	-	-	-	-	-	-	-	5.6	0	-	1.9
Pinaceae	*Pinus torreyana*	NH			-	-	-	-	-	-	-	-	2.8	0.1	0.3	1.1
Simaroubaceae	*Ailanthus altissima*			NK	-	-	-	-	-	-	-	-	2.8	0.3	0.1	1.0
Arecaceae	*Archontophoenix cunninghamiana*			NK	3.6	0	0.4	1.3	-	-	-	-	2.8	0	0.1	1.0
Asteraceae	*Baccharis pilularis*			NK	-	-	-	-	-	-	-	-	2.8	0.1	-	1.0
Arecaceae	*Phoenix canariensis*	NH			-	-	-	-	-	-	-	-	2.8	0	-	0.9
Cupressaceae	*Sequoia sempervirens*	NH			7.1	0.1	0.4	2.5	-	-	-	-	-	-	-	-
Sapindaceae	*Acer negundo*			K	3.6	0.1	2.6	2.1	-	-	-	-	-	-	-	-
Fagaceae	*Quercus lobata*			K	3.6	0	0.4	1.3	-	-	-	-	-	-	-	-
Anacardiaceae	*Schinus molle*		F		3.6	0	0	1.2	-	-	-	-	-	-	-	-
Total density (n*ha^{-1})					8,738				23,486				15,960			
Total Basal Area (m2ha^{-1})					134				786				588			

Supplementary Table 2. Output from the logistic regression testing the main effects of phylogenetically weighted host density (wpS), number of beetle generations (gens) and their interaction on FD–ISHB plot establishment. The regression takes the form logit(S) = β_0 + (β_1 x wpS) + (β_2 x gens) + (β_3 x [wpS x gens]) with the complementary log-log link function (AIC=229.68).

	**Coefficients**	**Standard Error**	***z*-Value**	**P-Value**
**Intercept**	-8.06	2.10	-3.84	0.0001
**Density-based wpS**	8.76	2.83	3.09	0.002
**Gens**	0.51	0.25	2.03	0.043
**wpS x Gens**	-0.80	0.34	-2.36	0.018
