# Supplementary material for: A phylogenetic epidemiology approach to predicting the establishment of multi-host plant pests
Source: Commun Biol. 2025 Jan 24;8:117. doi: 10.1038/s42003-025-07540-y (PMC11760915; doi:10.1038/s42003-025-07540-y)
Supplement: Supplementary file 2 — Description of Additional Supplementary File [file 42003_2025_7540_MOESM2_ESM.pdf]

Description Of Additional Supplementary File

File name: Supplementary Data
Description: Excel file containing Supplementary Data 1-9, which are source data behind the figures and data analyses in the paper. Supplementary Data 1 were used to perform the logistic regression in Fig. 2 and produce Fig. 3 and Supplementary Fig. 3. Supplementary Data 1, 4, and 5 were used to produce the map in Fig. 2, and to produce Fig. 4 and Supplementary Fig. 5. Supplementary Data 5 were used to produce Fig. 5. Supplementary Data 6-9 were used to develop the model.

File name: Supplementary Data 1
Description: Vegetation group, plot size, tree basal area (BA) and density, attacked tree density in 2017 and 2018, species richness, density- and basal area-based phylogenetically weighted susceptibility estimates, and ISHB generation estimates for each plot in Ventura, Orange, and San Diego Counties. Plots are presented in order from northwest to southeast within each county and vegetation group (California Broadleaf Forest & Woodland [BFW]; Semi-natural [SN]; and Southwestern North American Riparian Woodland, Forest, or Wash Scrub [RW, RF, or WS]).

File name: Supplementary Data 2.
Description: Total number of grids with FD–ISHB grid susceptibility estimates within each city in California.

File name: Supplementary Data 3.
Description: Urban forest tree species list used in this study to estimate FD–ISHB site susceptibility.

File name: Supplementary Data 4.
Description: ISHB generation estimates using degree-day models and wPS estimates using individual tree data from the California Urban Forest Inventory, aggregated to 1-km2 grids in 170 cities across the extent of California.

File name: Supplementary Data 5.
Description: Subset of 832 grids that were independently monitored for FD–ISHB across its known infested and non-infested geographic range and used to test the model.

File name: Supplementary Data 6.
Description: Phylogenetic distance matrix of all potential species ISHB could encounter calculated using a dated ultrametric phylogenetic tree developed by Lynch et al. (2021).

File name: Supplementary Data 7.
Description: Fusarium dieback-invasive shot hole borer host traits as described in Lynch et al. (2021).

File name: Supplementary Data 8.
Description: Species density by plot matrix used to calculate wpS.

File name: Supplementary Data 9.
Description: Species basal area by plot matrix used to calculate wpS.
